# Three-Dimensional Chiral Metal–Organic Frameworks: Synthesis and Structural Transformations

**DOI:** 10.3390/nano16010022

**Published:** 2025-12-24

**Authors:** Vadim A. Dubskikh, Anna A. Lysova, Denis G. Samsonenko, Konstantin A. Kovalenko, Danil N. Dybtsev, Vladimir P. Fedin

**Affiliations:** Nikolaev Institute of Inorganic Chemistry, Siberian Branch of Russian Academy of Sciences, 3 Acad. Lavrentiev Ave., Novosibirsk 630090, Russia

**Keywords:** metal–organic framework, chiral coordination polymers, structural transformation, benzene and cyclohexane separation

## Abstract

Four new porous homochiral metal–organic frameworks (MOFs), [M_2_(camph)_2_(bpa)]∙Solv (M = Co(II), Ni(II), Cu(II) and Zn(II)), based on (+)-camphoric acid (H_2_camph) and 1,2-bis(4-pyridyl)ethane (bpa) were synthesized and characterized. The crystal structures of [Ni_2_(camph)_2_(bpa)] and [Zn_2_(camph)_2_(bpa)] were established by single-crystal X-ray diffraction analysis. Powder X-ray data prove the phase purity and isostructural nature of all four compounds. The thermal stability of [M_2_(camph)_2_(bpa)] was found to depend on the electronic configuration, as well as on the redox properties of the metal cation, and varied from 225 °C (M = Zn^2+^) to 375 °C (M = Ni^2+^). The reversible, solvent-induced sponge-like dynamics of the coordination frameworks was thoroughly investigated. Changes in the positions of reflexes, related to the length of the flexible bpa linker, were observed by powder XRD, pointing to transitions between an open-framework phase and a squeezed, non-porous phase in a crystal-to-crystal manner, while the integrity and connectivity of the coordination network were maintained. Size-selective adsorption from a benzene–cyclohexane 1:1 mixture on [Zn_2_(camph)_2_(bpa)] was studied by ^1^H NMR analysis. The benzene-favorable composition of guest molecules (C_6_H_6_:C_6_H_12_ = 5:1) occluded within the host crystalline sponge revealed a preferable adsorption affinity towards smaller benzene compared with larger cyclohexane. High framework stability in various solvents, as well as successful molecular separation in the liquid state, validates the potential utilization of chiral porous metal(II) camphorate MOFs in important stereoselective applications.

## 1. Introduction

More than two decades of rapidly growing development of metal–organic frameworks prove that this class of compounds offers the greatest opportunities in terms of structural and functional design of porous materials. In particular, stereoselective (chiral) porous materials are among the most demanding areas in which MOFs are considered to be the most suited [1,2,3]. Indeed, enantio- and size-selective catalysis [4,5,6], separation of racemic mixtures and fine purification of chiral isomers [7,8,9] can readily be performed on such types of compounds. The corresponding processes are necessary for production of pharmaceuticals, many of which are chiral; therefore, the highest possible enantiopurity of a specific isomer is strictly required. Among other synthetic methods, a modular approach towards chiral porous MOFs using multiple or organic ligands has proved to be very convenient since it allows for independent variation in the size and geometry of the pores, as well as the chiral environment [10]. Also, such an approach may utilize readily available and naturally chiral molecules in MOF synthesis, affording valuable products at large scales without the necessity for complex organic synthesis. Despite obvious achievements [11,12,13,14,15,16,17], porous enantiopure MOFs are still greatly underdeveloped compared with their non-chiral counterparts; therefore, successful synthesis and characterization of new chiral frameworks are of substantial academic and practical significance. Specifically, the development of convenient synthetic methods for series of isostructural chiral porous MOFs is the most important since such compounds allow for the fine-tuning of porosity, chirality or other structural properties of the framework to best match the specific chiral substrate to be purified or catalyzed. Structural flexibility is another unique feature of many porous MOFs compared with zeolites or other rigid crystalline materials [18]. Apart from fundamental interest, such compounds afford significant advantages in molecular recognition [19], separation [20,21] and sensing [22] applications due to their ability to adapt the shape and size of the pores for more intimate host–guest interactions with the target substrate molecules.

Several years ago our group successfully introduced two series of isostructural porous enantiopure MOFs based on chiral lactic acid [23], mandelic acid [24] or camphoric acid [25,26], which possesses a rigid structure. The current report expands such series by four new open-framework chiral coordination polymers [M_2_(camph)_2_(bpa)], based on (+)-camphoric acid, auxiliary flexible linker 1,2-bis(4-pyridyl)ethane and late 3d metals M = Zn(II), Cu(II), Ni(II) and Co(II), prepared by modular one-pot synthesis. The thermal stability and solvent-induced dynamic behavior of coordination networks were also investigated in detail. The latter is particularly interesting and important as a processing of chiral molecules, where fine purification and catalytic activation are typically carried out in the liquid phase; therefore, the stability of porous material in various solvents has to be confirmed. Finally, the size-selective separation of a mixture of hydrocarbons was demonstrated, which additionally certifies the title homochiral porous MOFs for important future applications.

## 2. Materials and Methods

### 2.1. Instruments and Methods

The reagents were at least of reagent grade and used as purchased without additional purification. Infrared spectra of solid samples as KBr pellets were recorded using an IR-Fourier spectrometer Scimitar FTS 2000 (4000–400 cm^−1^) (Digilab LLC, Canton, MA, USA), providing an effective spectral resolution of 1 cm^−1^. The elemental analyses were obtained using «Vario Micro-Cube» (Elementar, Langenselbold, Germany) and «Euro EA 3000» (Eurovector Instruments, Pavia, Italy) analyzers. The thermogravimetric analyses were carried out in He atmosphere using a TG 209 F1 thermoanalyzer (NETZSCH, Selb, Germany) with a constant heating rate of 10 deg·min^–1^ in the temperature range from 25 °C to 600 °C. Powder X-ray diffraction data were obtained on a « TD-3700» powder diffractometer (Cu-Kα irradiation, λ = 1.54178 Å) (Tongda, Dandong, China) in the 2*θ* range from 5º to 30º. The ^1^H NMR spectra were recorded on an Avance 500 NMR spectrometer (Bruker, Karlsruhe, Germany). Optical photographs of the MOF samples were obtained on an MC2 Zoom 2CR microscope (Micromed, Saint Petersburg, Russia) equipped with an industrial digital camera ToupCam (Toup Tek Photonics, Hangzhou, China). SEM images of the MOF samples were obtained with a scanning electron microscope “S-3400N” (Hitachi, Tokyo, Japan) in secondary electrons. The accelerating voltage of the primary beam was 20 keV. The spraying of the conductive layer was not carried out. The porous structure was analyzed using the nitrogen adsorption technique on an Autosorb iQ gas sorption analyzer (Quantachrome, Boynton Beach, FL, USA) at 77 K. The preliminary activation of **1-Ni** was performed in the following way: The required amount of the MOF was immersed in 5 mL of THF for 5 days. Each day, the supernatant was decanted, and a new portion of acetone was added to the crystals. Then, the crystals were separated by decantation of the supernatant and dried under vacuum. The next step of activation was performed in a dynamic vacuum directly in the gas sorption analyzer.

### 2.2. X-Ray Crystallography

Single-crystal X-ray diffraction data for **1-Zn** and **1-Ni** were collected at 250 and 220 K, respectively, using a D8 Venture diffractometer equipped with a CMOS PHOTON III detector and an IμS 3.0 source (λ(MoKα) = 0.71073 Å for **1-Zn** and λ(CuKα) = 1.54178 Å for **1-Ni**; φ- and ω-scans) (Bruker, Karlsruhe, Germany). Absorption corrections were applied using SADABS [27]. The structures were solved by a dual-space algorithm (SHELXT [28]) and refined by the full-matrix least squares technique (SHELXL [29]) in the anisotropic approximation (except hydrogen atoms). Positions of hydrogen atoms in organic ligands were calculated geometrically and refined in the riding model. The crystallographic data and details of the structure refinements are summarized in Table S1. The structure contains a large void volume occupied with highly disordered DMF and H_2_O guest molecules, which could not be refined as a set of discrete atomic positions. The final composition of compounds **1-Zn** and **1-Ni** was defined according to the PLATON/SQUEEZE procedure [30] (300 *e*^−^ in 1264 Å^3^ for **1-Zn** and 375 *e*^−^ in 1229 Å^3^ for **1-Ni**) and the data from element (C, H and N) analyses. CCDC 2512318-2512319 contains the supplementary crystallographic data for this paper. These data can be obtained free of charge from The Cambridge Crystallographic Data Center at https://www.ccdc.cam.ac.uk/structures/ (accessed on 2 December 2025). The crystal data and structure refinement parameters for **1-Zn** and **1-Ni** are shown in Table S1.

### 2.3. Synthesis of Metal–Organic Coordination Polymers

**Synthesis of [Zn_2_(camph)_2_(bpa)]·3DMF·3H_2_O.** Zinc(II) nitrate hexahydrate (29.7 mg, 0.1 mmol; Vekton, Saint Petersburg, Russia), (+)-camphoric acid (H_2_camph; 20.0 mg, 0.1 mmol; Fluka, Burlington, MS, USA), 1,2-bis(4-pyridyl)ethane (bpa; 9.2 mg, 0.05 mmol; Sigma-Aldrich, Burlington, MS, USA) and 2.5 mL of N,N-dimethylformamide (DMF; Vekton, Saint Petersburg, Russia) were placed in a glass vial with a screw cap. The reaction mixture was sonicated for 30 min and then heated at 100 °C for 4 days. The resulting crystals were washed with DMF (3 × 5 mL) and dried in air. Yield 32 mg (65%). Anal. Calc. for [Zn_2_(camph)_2_(bpa)]·4DMF·2H_2_O (C_44_H_76_N_6_O_14_Zn_2_) (%): C 50.8, H 7.3, N 8.1%. Found: C 50.8, H 7.1, N 8.1%. IR data (cm^−1^): 508 (w), 555 (w), 659 (m), 804 (m), 1028 (w), 1091 (m), 1220 (w), 1293 (w), 1403 (s), 1616 (s), 1668 (s), 2879 (w), 2936 (w), 2968 (m), 3367 (w, broad).

**Synthesis of [Cu_2_(camph)_2_(bpa)]·2.4DMF·1.5H_2_O.** Copper(II) nitrate trihydrate (24.2 mg, 0.1 mmol; Vekton, Saint Petersburg, Russia), (+)-camphoric acid (H_2_camph; 20.0 mg, 0.1 mmol), 1,2-bis(4-pyridyl)ethane (bpa; 9.2 mg, 0.05 mmol), 1.25 mL of N,N-dimethylformamide (DMF) and 1.25 mL of ethanol were placed in a glass ampoule. The reaction mixture was sonicated for 30 min and then heated at 100 °C for 3 days. The resulting crystals were washed with DMF (3 × 5 mL) and dried in air. Yield 22.2 mg (48%). Anal. Calc. for [Cu_2_(camph)_2_(bpa)]·2.4DMF·1.5H_2_O (Cu_2_C_39.2_H_59.8_N_4.4_O_11.9_) (%): C 51.8, H 6.6, N 6.8%. Found: C 51.8, H 6.7, N 6.9%. IR data (cm^−1^): 413 (w), 528 (w), 575 (w), 670 (w), 804 (m), 1018 (w), 1112 (m), 1257 (w), 1294 (w), 1403 (s), 1616 (s), 1662 (s), 2879 (w), 2931 (m), 2963 (m), 3389 (m, broad).

**Synthesis of [Ni_2_(camph)_2_(bpa)]·4DMF·3H_2_O.** Nickel(II) nitrate hexahydrate (72.8 mg, 0.25 mmol; Vekton, Saint Petersburg, Russia), (+)-camphoric acid (H_2_camph; 50.0 mg, 0.25 mmol), 1,2-bis(4-pyridyl)ethane (bpa; 23 mg, 0.125 mmol) and 2.5 mL of N,N-dimethylformamide (DMF) were placed in a glass vial with a screw cap. The reaction mixture was sonicated for 30 min and then heated at 100 °C for 4 days. The resulting crystals were washed with DMF (3 × 5 mL) and dried in air. Yield 57.7 mg (45%). Anal. Calc. for [Ni_2_(camph)_2_(bpa)]·4DMF·1.5H_2_O (C_44_H_75_N_6_O_13.5_Ni_2_) (%): C 51.8, H 7.4, N 8.2%. Found: C 51.8, H 7.1, N 8.1%. IR data (cm^−1^): 518 (w), 602 (w), 654 (w), 804 (m), 1022 (w), 1095 (m), 1220 (w), 1288 (w), 1403 (s), 1616 (s), 1662 (s), 2874 (w), 2922 (w), 2962 (w), 3436 (w, broad).

**Activation of [Ni_2_(camph)_2_(bpa)]·4DMF·H_2_O.** Preliminary activation of **1-Ni** was performed in the following way: The required amount of the MOF was immersed in 5 mL of CH_2_Cl_2_ (Vekton, Saint Petersburg, Russia) or tetrahydrofuran (THF; Vekton, Saint Petersburg, Russia) for 5 days. The supernatant was decanted, and a new portion of THF was added to the crystals in a daily fashion. Then, the crystals were separated by decantation of the supernatant and dried under vacuum. The final step of activation was performed in a dynamic vacuum at 150 °C for 6 h in the gas adsorption analyzer.

**Synthesis of [Co_2_(camph)_2_(bpa)]·3.4DMF·4.8H_2_O.** Cobalt(II) nitrate hexahydrate (29.1 mg, 0.1 mmol; Vekton, Saint Petersburg, Russia), (+)-camphoric acid (H_2_camph; 20.0 mg, 0.1 mmol), 1,2-bis(4-pyridyl)ethane (bpa; 9.2 mg, 0.05 mmol), 1.75 mL of N,N-dimethylformamide (DMF) and 0.75 mL of methanol were placed in a glass ampoule. The reaction mixture was sonicated for 30 min and then heated at 100 °C for 3 days. The resulting crystals were washed with DMF (3 × 5 mL) and dried in air. Yield 45.8 mg (91%). Anal. Calc. for [Co_2_(camph)_2_(bpa)]·3.4DMF·4.8H_2_O (C_42.2_H_73.4_N_5.4_O_16.2_Co_2_) (%): C 49.0, H 7.1, N 7.3%. Found: C 48.9, H 7.2, N 7.3%. IR data (cm^−1^): 415 (w), 519 (w), 654 (w), 706 (w), 794 (m), 1033 (w), 1091 (m), 1174 (w), 1221 (w), 1288 (w), 1398 (s), 1506 (w), 1611 (s), 1678 (s), 2879, 2931 (m), 2968 (m), 3472 (w, broad).

### 2.4. Liquid-Phase Separation Experiments

In a typical experiment, as-synthesized **1-Zn** (0.100 g) was placed in a closed vial containing 10 mL of a 1:1 (*v*/*v*) benzene–cyclohexane mixture for 5 days. Then, the crystals were very filtered, quickly washed with two 5 mL portions of methanol and transferred into a vial where 0.7 mL of d_6_-dimethyl sulfoxide (DMSO-d_6_) and several drops of concentrated HCl were added. The mixture was sonicated for 10 min. The solution was transferred into a 5 mm NMR tube, and an ^1^H NMR spectrum of a mixture was recorded. The ratio of benzene and cyclohexane in the mixture was determined from the ratio of the integrals of the peaks corresponding to benzene (7.3–7.4 ppm) and cyclohexane (1.4 ppm), taking into account the number of protons.

## 3. Results and Discussion

The syntheses of all title compounds were optimized to achieve appreciable yields and crystallinity through a slight variation in reagent concentrations, as well as solvent compositions.

**Figure 1:**
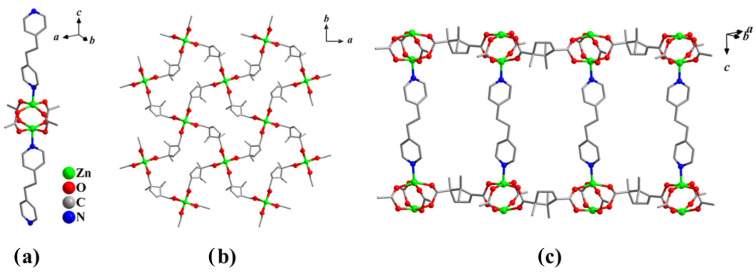
Crystal structure of **1-Zn**. (**a**) View of the {Zn_2_(OOCR)_4_} paddlewheel units with bpa ligands; (**b**) structure of the {Zn_2_(camph)_2_} layer; (**c**) projection of the framework along the {Zn_2_(camph)_2_} layers.

Colorless block crystals of the compound [Zn_2_(camph)_2_(bpa)]·3DMF·3H_2_O (**1-Zn**) were isolated in a solvothermal reaction of zinc(II) nitrate, H_2_camph and bpa (2:2:1 molar ratio) in N,N-dimethylformamide (DMF) at 100 °C. According to single-crystal X-ray diffraction data, **1-Zn** crystallizes in the tetragonal chiral space group *P*42_1_2. The asymmetric unit contains two crystallographically independent Zn(II) atoms with the square-pyramidal coordination environment of four oxygen atoms of four bridging carboxylate groups and one nitrogen atom of the bpa molecule in the axial position (Figure 1a). The Zn–O bond lengths are in the range 2.080(17)–2.156(13) Å, and the Zn–N distances are 2.048(7) Å. Two Zn ions form a binuclear “paddlewheel” complex {Zn_2_(RCOO)_4_}, connected by four disordered camph^2−^ anions into squeezed-square-grid layers (Figure 1b), which are further bound by the bpa linkers to form a 3D framework with a primitive cubic topology (**pcu**). The structure contains a two-dimensional system of intersecting channels running along the [110] and [1 $\bar{1}$0] directions and having the aperture of 10 × 5 Å (Figure 1c). The guest-accessible void volume of **1-Zn** is estimated by PLATON software (V-170925) [30] to be 55%.

It is to be noted that the {Zn_2_(camph)_2_} layers in crystal structure **1-Zn** are stacked exactly atop each other in AAAA mode. Earlier, in a similar isoreticular MOFs [Zn_2_(camph)_2_L] (L = diazabicyclo[2.2.2]octane, 4,4′-bipyridine) having shorter N-donor ligands (dabco and bpy, respectively), the packing mode of the {Zn_2_(camph)_2_} layers was established as AAAA in [Zn_2_(camph)_2_dabco] and alternating ABAB packing in [Zn_2_(camph)_2_bpy] [25]. We note that all these Zn(II)-camphorate chiral MOFs [Zn_2_(camph)_2_L] are crystallized from very similar conditions by the solvothermal reaction in DMF, with the length and nature of the auxiliary linker (L) being the only substantial difference. Peculiarly enough, the packing mode of the {Zn_2_(camph)_2_} layers alternates as AAAA ⟶ ABAB ⟶ AAAA when the length of the linker is continuously increased from dabco to bpy and, further, to bpa. Clearly, the fundamental factors affecting an important structural feature, such as the {Zn_2_(camph)_2_} packing mode in chiral MOFs, are yet to be uncovered, but every structurally characterized MOF greatly helps solve this puzzle.

**Figure 2:**
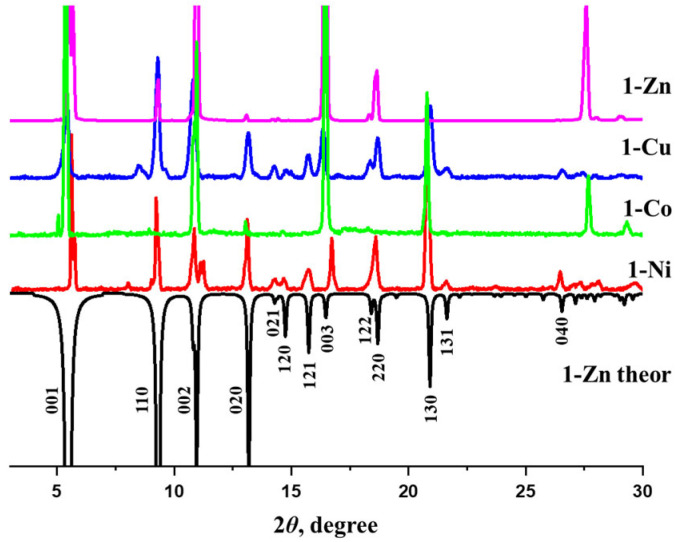
The PXRD patterns of as-synthesized **1-Zn**, **1-Ni**, **1-Co** and **1-Cu** in comparison with the theoretical **1-Zn** (black inverted line).

Small green block crystals of compound **1-Ni** were obtained under conditions similar to those used for compound **1-Zn**. Both compounds are isostructural (Table S1), but the unit cell parameters of **1-Ni** are slightly smaller compared with **1-Zn**. The coordination metal–ligand bond lengths are 1.096(3) ÷ 2.108(19) Å for Ni–O and 2.011(8) Å for Ni–N. Similarly to **1-Zn**, the disordered solvent molecules could not be located in the electron density map; however, the guest composition of **1-Ni** was derived from the PLATON/SQUEEZE procedure and microelemental CHN analysis. Unfortunately, our attempts to obtain single crystals of **1-Co** and **1-Cu** suitable for single-crystal X-ray diffraction experiments were unsuccessful. Nevertheless, powder X-ray diffraction data plainly showed that **1-Cu** and **1-Co** possess the same crystal structure as **1-Zn** and **1-Ni** (Figure 2). By indexing the most intense reflexes on the experimental XRD patterns, the following parameters of the tetragonal unit cell were identified: *a* = 13.36 Å and *c* = 16.17 Å for **1-Cu**; *a* = 13.79 Å and *c* = 16.48 Å for **1-Co**. The nature and composition of the solvent guest molecules in **1-Cu** and **1-Co** were established by a combination of chemical analysis, TGA and FT-IR spectroscopy.

### 3.1. Characterization of the Compounds

While the PXRD data (Figure 2) verify the phase purity of the synthesized compounds, the results of the chemical analyses and spectral data confirm their chemical purity and guest composition. As long as the IR spectra of all the title compounds feature the same characteristic bands (Figure S1), the detailed description is provided for prototypic **1-Zn** only. The medium-intensity band at 804 cm^−1^ is related to the non-planar deformations of the C–H bonds in the pyridine fragment of the bpa molecule. The medium-intensity bands at 1028 cm^−1^ correspond to the stretching of the C–N bond. The characteristic band at 1293 cm^−1^ is related to the symmetric stretching vibrations of the carboxylate groups. The strong band of the asymmetric stretching vibrations of the C=O bond in the carboxylate groups is observed at 1616 cm^–1^. The band at 1668 cm^−1^ can be ascribed to C=O bonds in DMF molecules. The higher energy peaks at 2968 cm^−1^ are assigned to the stretching vibrations of the C–H bonds in the methyl groups of the DMF molecule or camph^2–^ linker. The broad band at 3367 cm^−1^ corresponds to the valence vibrations of the O–H bond of guest water molecules.

**Figure 3:**
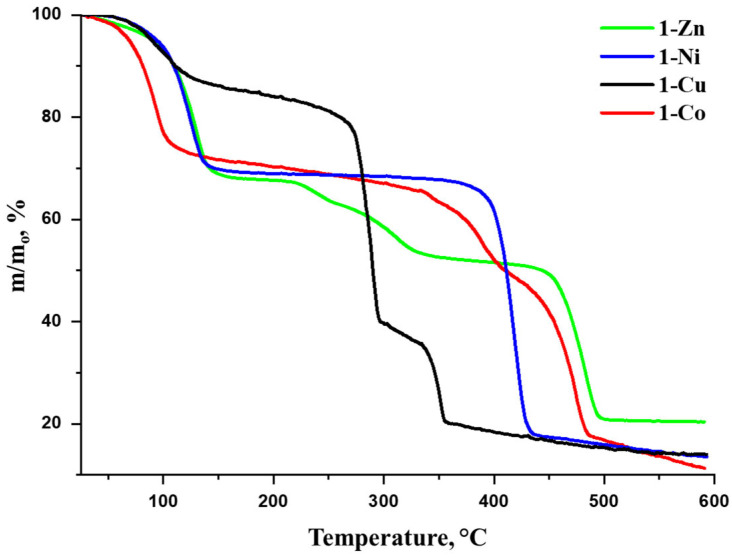
Thermogravimetric curves for compounds **1-Zn**, **1-Ni**, **1-Co** and **1-Cu**.

The thermal stability is an essential characteristic of the porous material proposed for separation applications. Despite very similar crystal structures and chemical compositions, the synthesized MOFs exhibit rather different behavior upon heating (Figure 3), except for a lower-temperature region (120–145 °C) where the loss of the guest solvent molecules takes place (boiling point of DMF is 153 °C), followed by a plateau. Up to this stage, the observed mass losses correspond to the proposed guest composition of the title MOFs: ~28% for **1-Co** (calculated: 27% for 3DMF and 3H_2_O),~31 wt.% for **1-Ni** (calculated: 31% for 4DMF and 1H_2_O), ~16 wt.% for **1-Cu** (calculated: 15% for 1DMF and 3H_2_O) and ~33 wt.% for **1-Zn** (calculated: 35% for 4DMF and 4H_2_O). The lower guest composition of **1-Cu**, evidenced by the chemical and thermogravimetric analyses, should probably be attributed to premature solvent evaporation during sample handling since the size of the crystallites of **1-Cu** is smaller (Figure S12), compared with the other compounds of the series. The stability of the guest-free [M_2_(camph)_2_bpa] framework at higher temperatures depend strongly on the nature of the metal cations. Particularly, the MOF stability is limited either by M(II) oxidation potential or by the strength of the metal(II)–ligand coordination bonds. The standard oxidation potentials *E*^0^(M^2+^/M) are known to be *E*^0^ = –0.76 V (Zn), –0.29 V (Co), –0.26 V (Ni) and +0.35 V (Cu); therefore, due to moderate oxidation properties of the Cu(II) cations, compound **1-Cu** shows rather low decomposition temperature. The other cations have little or no redox activity; hence, the thermal behavior of **1-Co**, **1-Ni** and **1-Zn** mostly correlates with the stability of the coordination complexes of these metal cations according to the empirical Irving–Williams series. In general, the stability of complexes is increased for late 3*d* transition metals due to a decrease in the ionic radii and an increase in the crystal field stabilization energy of the partially deficient d-orbitals. However, as soon as the d-orbitals become fully occupied in case of the Zn(II) cation, the stability of the corresponding complexes drops sharply due to the absence of any electronic stabilization of the *d*^10^ electronic configuration. With the exception of **1-Cu**, whose thermal stability is limited due to redox properties (*T*~270 °C), the observed TGA data for the title MOFs follow the Irving–Williams empirical rule. Indeed, the framework decomposition temperature is increased from **1-Co** (340 °C*, d*^7^ cation) to **1-Ni** (375 °C, *d*^8^ cation) and drops for **1-Zn** (225 °C, *d*^10^ cation). The reported findings represent a rare demonstration of the customization of the stability of isostructural MOFs through a variation in the metal cations while the size and geometry of the chiral pores of the frameworks are maintained the same.

### 3.2. Structural Transformations

**Figure 4:**
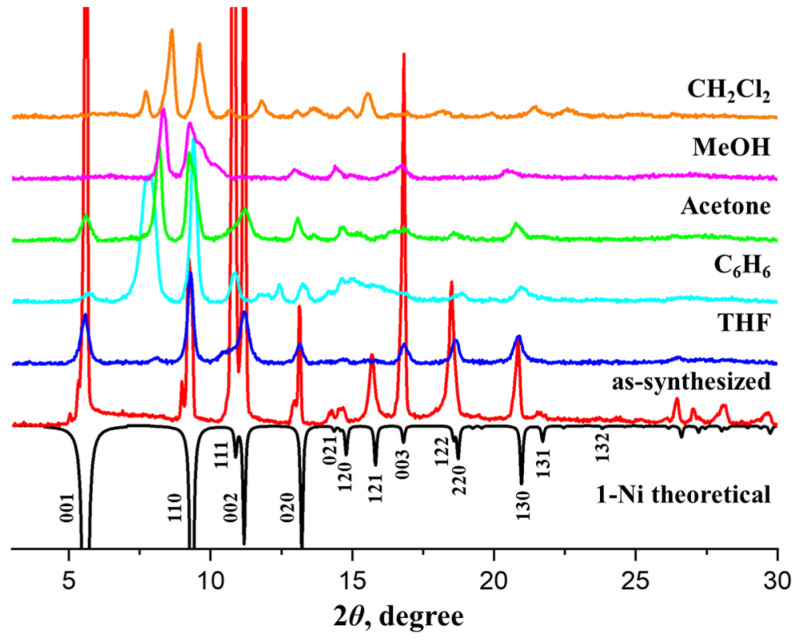
Powder XRD patterns of **1**-N**i** crystalline sample kept in different low-boiling solvents.

The remarkable guest-accessible volume of two-dimensional channels in **1** encouraged an investigation of the guest adsorption properties, as well as the framework stability upon solvent substitution. Based on the TGA data, compound **1-Ni**, featuring the broadest temperature stability range due to stronger metal–ligand coordination bonds, was a primary choice for such experiments. The as-synthesized crystals of **1-Ni** were soaked in various solvents (dichloromethane, methanol, benzene, acetone and tetrahydrofuran) for five days to exchange the DMF/H_2_O guest molecules. The powder XRD pattern of the solvent-exchanged **1-Ni⸧Solv** (Figure 4) indicated substantial structural changes in the framework for all solvents except for tetrahydrofuran. While the peak at 2θ ≈ 9.5°, related to the Ni-camph-Ni distance through the rigid camphorate linkers, is retained, new strong reflexes emerged at 2θ ≈ 7.9° ÷ 8.6°, which correspond to the interplanar distances *d* ≈ 11.2 ÷ 10.3 Å, according to the Bragg diffraction formula *n*∙λ = 2∙*d*∙sinθ. Along with new reflexes, the strong peak at 2θ ≈ 5.5° related to the Zn-bpa-Zn distances *d* = 16.1 Å almost disappeared. Although we do not have unambiguous single-crystal X-ray diffraction data, the emergence of new reflexes at 2θ ≈ 7.9° ÷ 8.6° in **1-Ni⸧Solv** instead of 2θ ≈ 5.5° in **1-Ni⸧DMF** strongly suggests that the Ni-bpa-Ni distances are squeezed upon solvent exchange. Such significant structural distortion could be possible by taking the flexibility of the ethylene moiety of the bpa linker into account [31,32,33].

Most importantly, the original MOF structure could be restored when the solvent-exchanged samples **1-Ni⸧Solv** were immersed into DMF. The powder XRD pattern of regenerated **1-Ni** shows complete restoration of the original reflexes (Figures S2–S4). Some broadening of the diffraction peaks may refer to macroscopic defects of the crystallites invoked by the transformation of the organic ligands during solvent substitution. In spite of remarkable flexibility of the structure, both integrity and connectivity of the coordination network are preserved during the solvent exchange process, as clearly evidenced by the fully reversible transformations accomplished in crystal-to-crystal mode.

**Figure 5:**
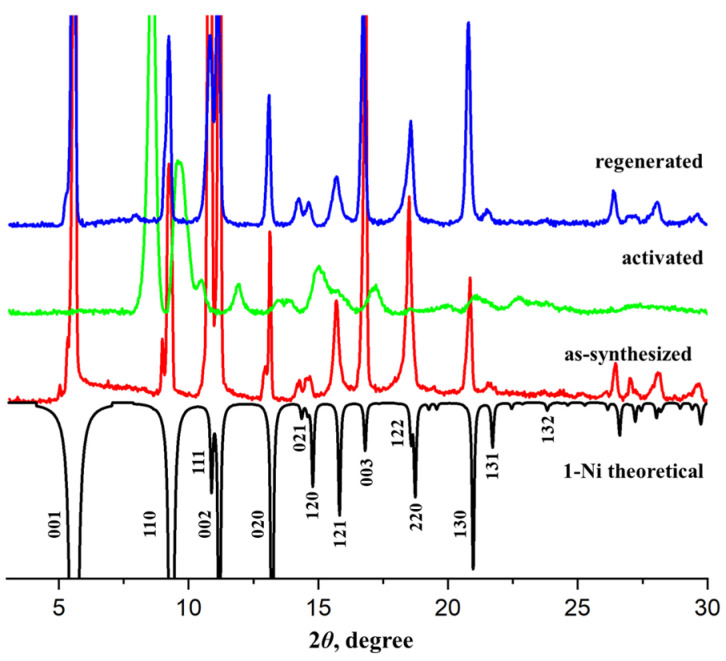
Powder XRD patterns of the as-synthesized **1-Ni** sample were recorded after it underwent direct vacuum activation at 100 °C for 4 h and was subsequently regenerated in DMF.

A number of attempts of activation of **1-Ni⸧CH_2_Cl_2_**, as well as other solvent-exchanged samples, in dynamic vacuum were carried out. Regardless of the activation conditions (starting from ambient temperature up to 150 °C), all experiments afforded non-porous products, according to the gas adsorption measurements. Similar results were obtained for **1-Ni⸧THF** despite the fact that the crystal structure of **1-Ni** is seemingly preserved upon solvent substitution in tetrahydrofuran (Figure S5). Finally, direct vacuum activation of as-synthesized **1-Ni** at 100 °C resulted in the same non-porous product with squeezed structure (Figure 5). The large free volume of the pristine framework seems to collapse upon guest removal since the flexible bpa linkers are not able to provide sufficient rigidity. Similar results were observed earlier for the [Zn_2_(camph)_2_bpe] (bpe = 1,2-*bis*(4-pyridil)ethylene) compound, which also collapsed upon activation [25]. While permanent porosity is absolutely essential to adsorption of small gases, catalytic activation and purification of larger chiral molecules are typically carried out in the liquid phase. In this regard, an investigation of the stability and solvent-induced structural transformations in the framework, as described above, is far more relevant and important. We also note that the guest-free non-porous phase is expanded back to the original open-framework structure of **1-Ni** when the vacuum-activated crystals are rejuvenated in DMF solvent (Figure 5), which also confirms the preservation of the integrity of the coordination framework during guest removal despite significant transformations in the crystal structure.

Other coordination polymers, **1-Co**, **1-Cu** and **1-Zn**, exhibit similar structural transformations to squeezed phase upon solvent exchange in methylene chloride and room-temperature vacuum activation. Powder XRD diffraction data indicate major changes in the positions of reflexes, related to the crystallographic *c* axis of the tetragonal unit cell, which, in turn, depends solely on the length and conformation of the flexible bpa organic linker (Figures S6–S8). Such transformations are fully reversible for **1-Zn** by immersing the activated crystals in DMF. On the contrary, the squeezed phases of guest-free **1-Co** and **1-Cu** could hardly be restored back to the original state in DMF solvent. According to the XRD data, only weak reflexes related to the pristine framework appear on the DMF-soaked **1-Co** sample, while **1-Cu** shows degradation of its crystallinity. It is to be noted that the optimized synthetic conditions for **1-Co** and **1-Cu** require the presence of alcohols in the reaction medium because the yield and/or crystallinity of the products got worse in pure DMF. Quite likely, the connectivity of the coordination network in **1-Co** and **1-Cu** during the solvent exchange and activation experiments is retained, but the restoration of the original open-framework conformation requires some tweaks in the solvent composition.

### 3.3. Adsorption Studies

As mentioned above, hydrolytic stability in different solvents and facile exchange of guest molecules are essential to chiral porous materials considered for important enantioselective applications. Demonstration of the adsorption and separation potential of the isostructural title MOFs was carried out on **1-Zn**, since it demonstrates high framework stability. Also, Zn(II) is a diamagnetic cation; therefore, the NMR technique could be used for quantitative determination of the analytes. Liquid-phase adsorption experiments were carried out by immersing crystals of **1-Zn** in a 1:1 (by volume) mixture of benzene and cyclohexane for several days to reach an equilibrium. The crystals were collected, rinsed and dissolved in DMSO-d_6_ with some amount of HCl. The composition of the organic guest molecules was analyzed by ^1^H NMR, which revealed a C_6_H_6_:C_6_H_12_ = 5:1 relative molar ratio, while the MOF–guest composition was ca. 11 wt.%, which roughly corresponds to the formula [Zn_2_(camph)_2_bpa]∙1.6(C_6_H_6_/C_6_H_12_) (Figure S9). The pronounced affinity of **1-Zn** towards aromatic benzene over aliphatic cyclohexane is surprising to a certain extent, taking a predominantly aliphatic nature of the pore environment of the camphorate framework into account. The smaller size of the C_6_H_6_ molecule (ca. 7.3 × 6.6 × 3.3 Å), compared with the C_6_H_12_ molecule (ca. 7.2 × 6.6 × 5.0 Å) [34], seems to be a plausible explanation for the observed C_6_H_6_ > C_6_H_12_ adsorption selectivity, especially if a narrow channel diameter in the squeezed **1-Zn** structure is assumed under the particular experimental conditions. Apparently, the **1-Zn** porous material behaves like a size-selective molecular sieve. The obtained C_6_H_6_/C_6_H_12_ selectivity factor (*S* = 5) surpasses that of many other MOF materials reported in the literature under similar conditions [35,36,37,38,39,40], although there are several exceptions with much higher C_6_H_6_/C_6_H_12_ selectivity [41,42,43]. The obtained results highlight promising application potential of **1-Zn** as well as the other metal camphorates described in the current work for separation of benzene from cyclohexane, which is an important step in the synthesis of caprolactam and the production of polyamide polymers like nylon. Moreover, successful molecular separation in liquid state validates the potential utilization of the metal(II) camphorate porous MOFs in important stereoselective applications.

## 4. Conclusions

The new homochiral porous metal(II)-camphorate metal–organic frameworks (MOFs) represent an important contribution to such a highly valuable sub-class of porous materials. The tunable stability and reversible sponge-like dynamics of the coordination frameworks and the demonstration of the remarkable size-selective adsorption of the MOFs provide a solid background for potential utilization of such materials in important industrial processes involving stereoselective purification of chiral molecules or separation of racemic mixtures.

## Data Availability

CCDC 2512318-2512319 contains the supplementary crystallographic data for this paper. These data can be obtained free of charge from The Cambridge Crystallographic Data Center at https://www.ccdc.cam.ac.uk/structures/ (2 December 2025).

## Supplementary Materials

The following supporting information can be downloaded at: https://www.mdpi.com/article/10.3390/nano16010022/s1, Figure S1: IR spectra of the obtained MOFs, Figure S2: The PXRD patterns of sample **1-Ni** placed in acetone, as-synthesized (red), activated (blue) and regenerated (green), compared with the theoretical one for **1-Ni** (black), Figure S3: The PXRD patterns of sample **1-Ni** placed in methanol, as-synthesized (red), activated (blue) and regenerated (green), compared with the theoretical one for **1-Ni** (black), Figure S4: The PXRD patterns of sample **1-Ni** placed in benzene, as-synthesized (red), activated (blue) and regenerated (green), compared with the theoretical one for **1-Ni** (black), Figure S5: The PXRD patters of **1-Ni** activated from tetrahydrofuran after adsorption experiments, Figure S6: The PXRD patterns of sample **1-Zn** activated from dichloromethane followed by vacuum treatment at room temperature for 1 h, Figure S7: The PXRD patterns of sample **1-Co** activated from dichloromethane followed by vacuum treatment at room temperature for 1 h, Figure S8: The PXRD patterns of sample **1-Cu** activated from dichloromethane followed by vacuum treatment at room temperature for 1 h, Figure S9: ^1^H NMR spectrum of benzene and cyclohexane desorbed from **1-Zn**, Figure S10: SEM images of **1-Co, 1-Cu, 1-Ni and 1-Zn** crystals, Figure S11: EDS mapping of **1-Ni** crystals, Figure S12: Optical images of **1-Zn, 1-Ni, 1-Co and 1-Cu** crystals, Figure S13: Nitrogen adsorption–desorption isotherms at 77 K for sample **1-Ni** activated from CH_2_Cl_2_ (left) and THF (right). The corresponding activation temperature is provided in the legend, Figure S14: The asymmetric units for compounds **1-Zn** (top) and **1-Ni** (bottom), Figure S15: Rietveld refinement of PXRD data for compound **1-Co** performed using Profex software (V5.6), Figure S16: Rietveld refinement of PXRD data for compound **1-Cu** performed using Profex software, Figure S17: Rietveld refinement of PXRD data for compound **1-Ni** performed using Profex software, Figure S18: Rietveld refinement of PXRD data for compound **1-Zn** performed using Profex software, Table S1: Crystal data and structure refinement for **1-Zn** and **1-Ni**, Table S2: Elemental composition of **1-Ni, 1-Zn, 1-Co and 1-Cu** determined by energy-dispersive X-ray spectroscopy.

## References

[1] Seo J., Whang D., Lee H., Jun S.I., Oh J., Jeon Y.J., Kim K. (2000). A homochiral metal-organic porous material for enantioselective separation and catalysis. Nature.

[2] Kepert C.J., Prior T.J., Rosseinsky M.J. (2000). A Versatile Family of Interconvertible Microporous Chiral Molecular Frameworks:  The First Example of Ligand Control of Network Chirality. J. Am. Chem. Soc..

[3] Ma L., Lin W. (2008). Chirality-Controlled and Solvent-Templated Catenation Isomerism in Metal–Organic Frameworks. J. Am. Chem. Soc..

[4] Gheorghe A., Tepaske M.A., Tanase S. (2018). Homochiral metal–organic frameworks as heterogeneous catalysts. Inorg. Chem. Front..

[5] Ma M., Chen J., Liu H., Huang Z., Huang F., Li Q., Xu Y. (2022). A review on chiral metal–organic frameworks: Synthesis and asymmetric applications. Nanoscale.

[6] Zhang H., Lou L.-L., Yu K., Liu S. (2021). Advances in Chiral Metal–Organic and Covalent Organic Frameworks for Asymmetric Catalysis. Small.

[7] Pacchioni G. (2023). A chiral supramolecular MOF for enantiomer separation. Nat. Rev. Mater..

[8] Fan Y., Chen M. (2025). Emerging frontiers in chiral metal–organic framework membranes: Diverse synthesis techniques and applications. Nanoscale.

[9] Liu J., Mukherjee S., Wang F., Fischer R.A., Zhang J. (2021). Homochiral metal–organic frameworks for enantioseparation. Chem. Soc. Rev..

[10] Kesanli B., Lin W. (2003). Chiral porous coordination networks: Rational design and applications in enantioselective processes. Coord. Chem. Rev..

[11] Deng C., Liu X., Wang Z., Lin W. (2025). Homochiral BINOL-Based Metal–Organic Frameworks for Luminescence Sensing of Hydrobenzoin Enantiomers. Inorg. Chem..

[12] Deng C., Song B.-Q., Sensharma D., Gao M.-Y., Bezrukov A.A., Nikolayenko V.I., Lusi M., Mukherjee S., Zaworotko M.J. (2023). Effect of Extra-Framework Anion Substitution on the Properties of a Chiral Crystalline Sponge. Cryst. Growth Des..

[13] Zheng X., Zhang Q., Ma Q., Li X., Zhao L., Sun X. (2023). A chiral metal-organic framework {(HQA)(ZnCl_2_)(2.5H_2_O)}_n_ for the enantioseparation of chiral amino acids and drugs. J. Pharm. Anal..

[14] Gheorghe A., Strudwick B., Dawson D.M., Ashbrook S.E., Woutersen S., Dubbeldam D., Tanase S. (2020). Synthesis of Chiral MOF-74 Frameworks by Post-Synthetic Modification by Using an Amino Acid. Chem. Eur. J..

[15] Zhao T., Han J., Shi Y., Zhou J., Duan P. (2021). Multi-Light-Responsive Upconversion-and-Downshifting-Based Circularly Polarized Luminescent Switches in Chiral Metal–Organic Frameworks. Adv. Mater..

[16] Liu Y., Liu L., Chen X., Liu Y., Han Y., Cui Y. (2021). Single-Crystalline Ultrathin 2D Porous Nanosheets of Chiral Metal–Organic Frameworks. J. Am. Chem. Soc..

[17] Gong W., Chen X., Fahy K.M., Dong J., Liu Y., Farha O.K., Cui Y. (2023). Reticular Chemistry in Its Chiral Form: Axially Chiral Zr(IV)-Spiro Metal–Organic Framework as a Case Study. J. Am. Chem. Soc..

[18] Behera N., Duan J., Jin W., Kitagawa S. (2021). The Chemistry and Applications of Flexible Porous Coordination Polymers. EnergyChem.

[19] Zhang S.-Y., Fairen-Jimenez D., Zaworotko M.J. (2020). Structural Elucidation of the Mechanism of Molecular Recognition in Chiral Crystalline Sponges. Angew. Chem. Int. Ed..

[20] Demakov P.A., Poryvaev A.S., Kovalenko K.A., Samsonenko D.G., Fedin M.V., Fedin V.P., Dybtsev D.N. (2020). Structural Dynamics and Adsorption Properties of the Breathing Microporous Aliphatic Metal–Organic Framework. Inorg. Chem..

[21] Deng C., Song B.-Q., Lusi M., Bezrukov A.A., Haskins M.M., Gao M.-Y., Peng Y.-L., Ma J.-G., Cheng P., Mukherjee S. (2023). Crystal Engineering of a Chiral Crystalline Sponge That Enables Absolute Structure Determination and Enantiomeric Separation. Cryst. Growth Des..

[22] Demakov P.A., Ryadun A.A., Dybtsev D.N. (2022). Highly Luminescent Crystalline Sponge: Sensing Properties and Direct X-ray Visualization of the Substrates. Molecules.

[23] Dybtsev D.N., Nuzhdin A.L., Chun H., Bryliakov K.P., Talsi E.P., Fedin V.P., Kim K. (2006). A Homochiral Metal–Organic Material with Permanent Porosity, Enantioselective Sorption Properties, and Catalytic Activity. Angew. Chem. Int. Ed..

[24] Dybtsev D.N., Yutkin M.P., Samsonenko D.G., Fedin V.P., Nuzhdin A.L., Bezrukov A.A., Bryliakov K.P., Talsi E.P., Belosludov R.V., Mizuseki H. (2010). Modular, Homochiral, Porous Coordination Polymers: Rational Design, Enantioselective Guest Exchange Sorption and Ab Initio Calculations of Host–Guest Interactions. Chem. Eur. J..

[25] Dybtsev D.N., Yutkin M.P., Peresypkina E.V., Virovets A.V., Serre C., Férey G., Fedin V.P. (2007). Isoreticular Homochiral Porous Metal–Organic Structures with Tunable Pore Sizes. Inorg. Chem..

[26] Dybtsev D.N., Yutkin M.P., Fedin V.P. (2009). Copper(II) camphorates with tunable pore size in metal-organic frameworks. Russ. Chem. Bull..

[27] (2017). Bruker Apex3 Software Suite: Apex3, SADABS-2016/2 and SAINT.

[28] Sheldrick G.M. (2015). SHELXT–Integrated space-group and crystal-structure determination. Acta Crystallogr. Sect. A.

[29] Sheldrick G.M. (2015). Crystal structure refinement with SHELXL. Acta Crystallogr. Sect. C.

[30] Spek A.L. (2015). PLATON SQUEEZE: A tool for the calculation of the disordered solvent contribution to the calculated structure factors. Acta Crystallogr. Sect. C.

[31] Bezuidenhout C.X., Smith V.J., Esterhuysen C., Barbour L.J. (2017). Solvent- and Pressure-Induced Phase Changes in Two 3D Copper Glutarate-Based Metal–Organic Frameworks via Glutarate (+gauche ⇄ −gauche) Conformational Isomerism. J. Am. Chem. Soc..

[32] Kim H.-C., Huh S., Kim J.Y., Moon H.R., Lee D.N., Kim Y. (2017). Zn-MOFs containing flexible α,ω-alkane (or alkene)-dicarboxylates with 1,2-bis(4-pyridyl)ethylene: Comparison with Zn-MOFs containing 1,2-bis(4-pyridyl)ethane ligands. CrystEngComm.

[33] Tahier T., Oliver C.L. (2017). A Cd mixed-ligand MOF showing ligand-disorder induced breathing behaviour at high temperature and stepwise, selective carbon dioxide adsorption at low temperature. CrystEngComm.

[34] Mukherjee S., Sensharma D., Qazvini O.T., Dutta S., Macreadie L.K., Ghosh S.K., Babarao R. (2021). Advances in adsorptive separation of benzene and cyclohexane by metal-organic framework adsorbents. Coord. Chem. Rev..

[35] Duan L., Wu Z.-H., Ma J.-P., Wu X.-W., Dong Y.-B. (2010). Adsorption and Separation of Organic Six-Membered Ring Analogues on Neutral Cd(II)-MOF Generated from Asymmetric Schiff-Base Ligand. Inorg. Chem..

[36] Zeng M.-H., Tan Y.-X., He Y.-P., Yin Z., Chen Q., Kurmoo M. (2013). A Porous 4-Fold-Interpenetrated Chiral Framework Exhibiting Vapochromism, Single-Crystal-to-Single-Crystal Solvent Exchange, Gas Sorption, and a Poisoning Effect. Inorg. Chem..

[37] Wang J.-H., Luo D., Li M., Li D. (2017). Local Deprotonation Enables Cation Exchange, Porosity Modulation, and Tunable Adsorption Selectivity in a Metal–Organic Framework. Cryst. Growth Des..

[38] Yao H., Wang Y.M., Quan M., Farooq M.U., Yang L.P., Jiang W. (2020). Adsorptive separation of benzene, cyclohexene, and cyclohexane by amorphous nonporous amide naphthotube solids. Angew. Chem. Int. Ed..

[39] Feng X., Hu D.Y., Liang Z.-J., Zhou M.Y., Wang Z.-S., Su W.-Y., Lin R.-B., Zhou D.-D., Zhang J.-P. (2025). A metal azolate framework with small aperture for highly efficient ternary benzene/cyclohexene/cyclohexane separation. Chin. J. Struct. Chem..

[40] Han Y., Chen Y., Ma Y., Bailey J., Wang Z., Lee D., Sheveleva A.M., Tuna F., McInnes E.J.L., Frogley M.D. (2023). Control of the pore chemistry in metal-organic frameworks for efficient adsorption of benzene and separation of benzene/cyclohexane. Chem.

[41] Lysova A.A., Samsonenko D.G., Dorovatovskii P.V., Lazarenko V.A., Khrustalev V.N., Kovalenko K.A., Dybtsev D.N., Fedin V.P. (2019). Tuning the molecular and cationic affinity in a series of multifunctional metal–organic frameworks based on dodecanuclear Zn(II) carboxylate wheels. J. Am. Chem. Soc..

[42] Sapianik A.A., Kovalenko K.A., Samsonenko D.G., Barsukova M.O., Dybtsev D.N., Fedin V.P. (2020). Exceptionally effective benzene/cyclohexane separation using a nitro-decorated metal–organic framework. Chem. Commun..

[43] Poryvaev A.S., Yazikova A.A., Polyukhov D.M., Fedin M.V. (2022). Ultrahigh selectivity of benzene/cyclohexane separation by ZIF-8 framework: Insights from spin-probe EPR spectroscopy. Microporous Mesoporous Mater..

