# Supplementary material for: Three-Dimensional Chiral Metal–Organic Frameworks: Synthesis and Structural Transformations
Source: Nanomaterials (Basel). 2025 Dec 24;16(1):22. doi: 10.3390/nano16010022 (PMC12787545; doi:10.3390/nano16010022)
Supplement: Supplementary file 1 [file nanomaterials-16-00022-s001.zip › nanomaterials-4050481-supplementary.pdf]

3D chiral metal-organic frameworks: synthesis and structural transformation

Vadim A. Dubskikh,[a] Anna A. Lysova,[a] Denis G. Samsonenko,[a] Konstantin A. Kovalenko,[a] Danil N. Dybtsev[a] and Vladimir P. Fedin[a]

[a]Nikolaev Institute of Inorganic Chemistry, Siberian Branch of Russian Academy of Sciences, 3 Acad. Lavrentiev Ave., Novosibirsk 630090, Russia

Table S1. Crystal data and structure refinement for **1-Zn** and **1-Ni**

Compound/parameter	**1-Zn**	**1-Ni**
Empirical formula	$C_{41}H_{67}N_5O_{14}Zn_2$	$C_{44}H_{74}N_6O_{15}Ni_2$
M, g/mol	984.73	1044.51
Crystal system	*Tetragonal*	*Tetragonal*
Space group	$P42_12$	$P42_12$
a, Å	13.4303(16)	13.3968(14)
b, Å	13.4303(16)	13.3968(14)
c, Å	16.1453(18)	15.826(2)
α, deg.	90	90
β, deg.	90	90
γ, deg.	90	90
V, Å^3	2912.2(8)	2840.4(7)
Z	2	2
D(calc.), г/см^3	1.123	1.221
μ, mm^{-1}	0.878	1.346
F(000)	1040	1112
Crystal size, mm	0.30 × 0.30 ×0.20	0.15 × 0.09 ×0.07
θ range for data collection, deg.	1.97 – 28.28	4.32 – 70.35
Index ranges *hkl*	$-17 \le h \le 17$,<br>$-17 \le k \le 17$,<br>$-21 \le l \le 12$,	$-16 \le h \le 16$,<br>$-15 \le k \le 16$,<br>$-19 \le l \le 18$,
Reflections collected / independent	30783 / 3603	18263 / 2714
R_{int}	0.0860	0.0653
Reflections with $I > 2\sigma(I)$	2115	1983
Goodness-of-the on F^2	0.915	0.972
Final *R* indices [$I > 2\sigma(I)$]	$R_1 = 0.0396$,<br>$wR_2 = 0.0904$	$R_1 = 0.0577$,<br>$wR_2 = 0.1500$
R indices (all data)	$R_1 = 0.0912$,<br>$wR_2 = 0.1006$	$R_1 = 0.0741$,<br>$wR_2 = 0.1601$
Largest diff. peak / hole, e/Å^3	0.888 / – 0.223	1.210 / – 0.321

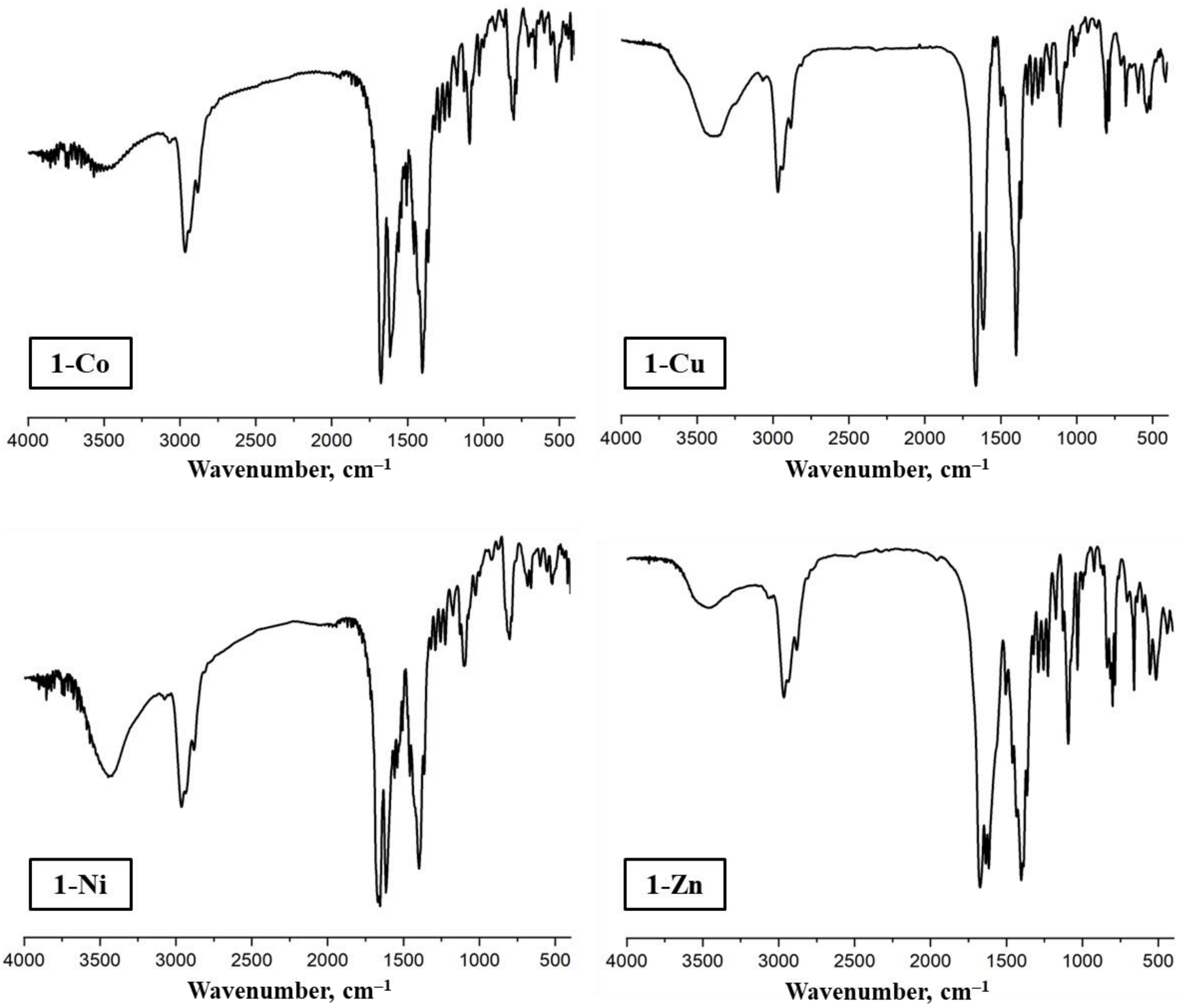

Figure S1. IR spectra of the obtained MOFs.

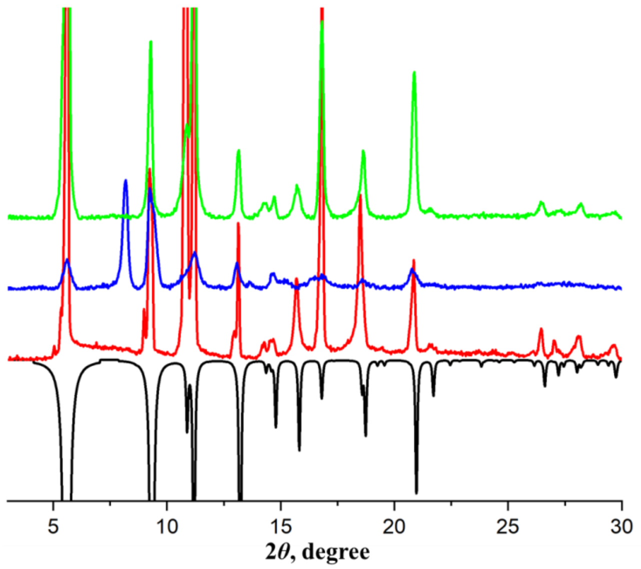

Figure S2. The PXRD patterns of the sample **1-Ni** placed in acetone: as-synthesized (red), activated (blue) and regenerated (green) compared to the theoretical one for the **1-Ni** (black).

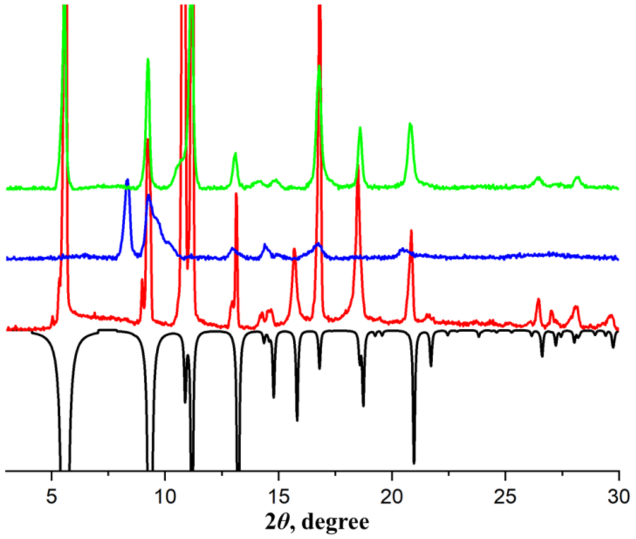

Figure S3. The PXRD patterns of the sample **1-Ni** placed in methanol: as-synthesized (red), activated (blue) and regenerated (green) compared to the theoretical one for the **1-Ni** (black).

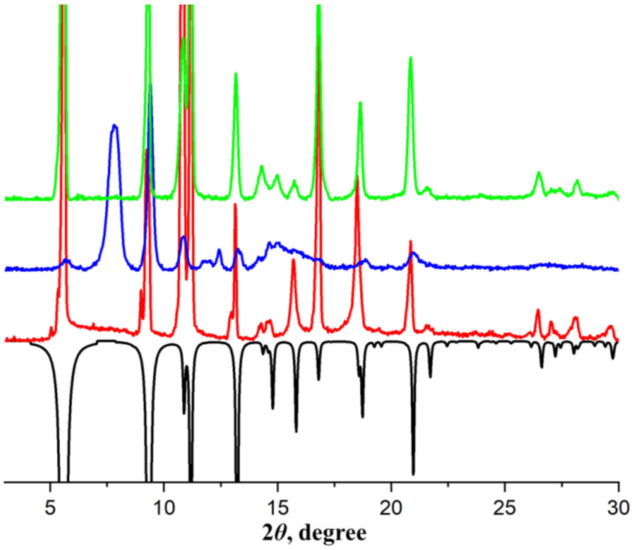

Figure S4. The PXRD patterns of the sample **1-Ni** placed in benzene: as-synthesized (red), activated (blue) and regenerated (green) compared to the theoretical one for the **1-Ni** (black).

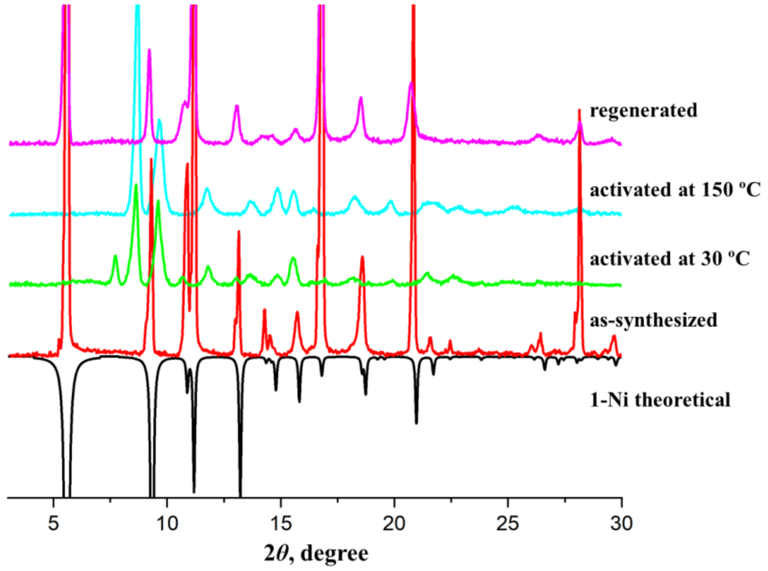

Figure S5. The PXRD patters of **1-Ni** activated from tetrahydrofuran after adsorption experiments.

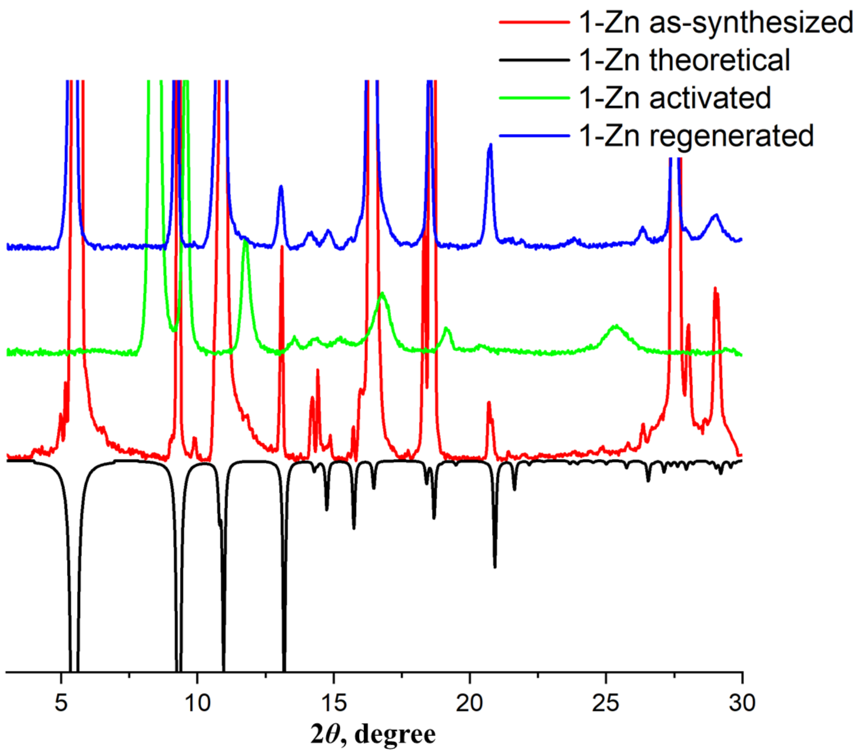

Figure S6. The PXRD patterns of the sample **1-Zn** activated from dichloromethane followed by vacuum treatment at room temperature for 1 hour.

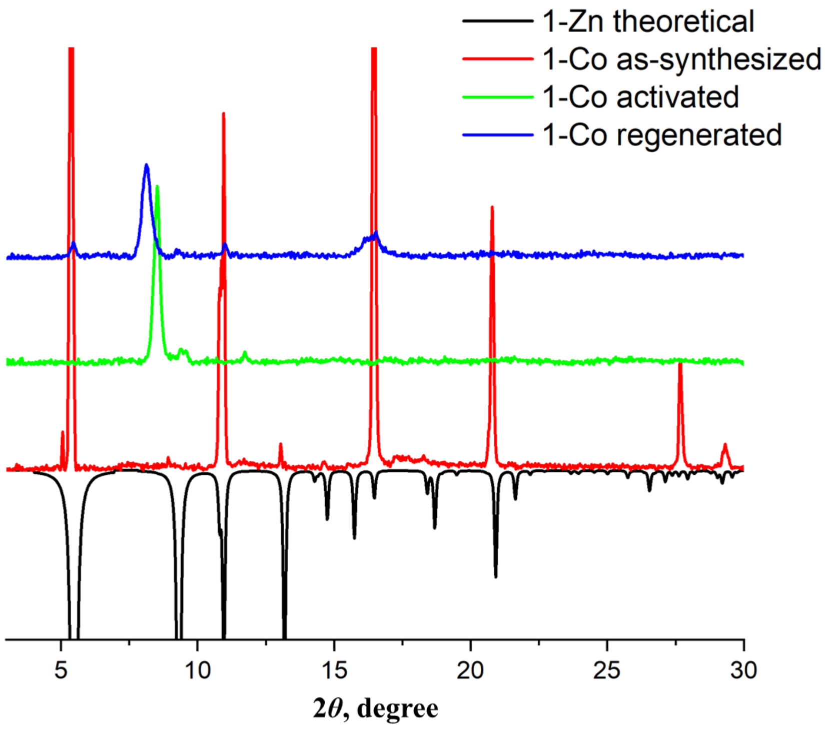

Figure S7. The PXRD patterns of the sample **1-Co** activated from dichloromethane followed by vacuum treatment at room temperature for 1 hour.

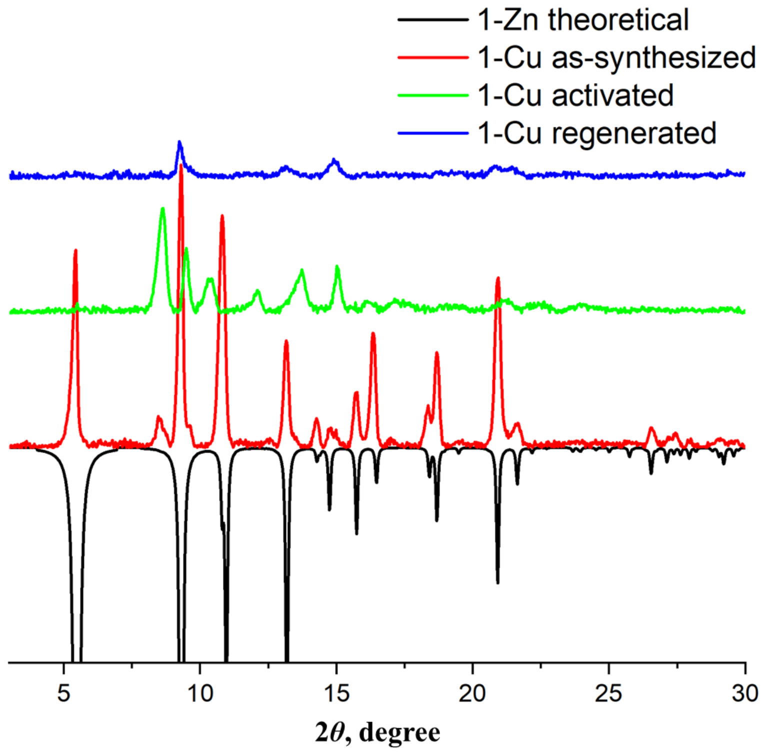

Figure S8. The PXRD patterns of the sample **1-Cu** activated from dichloromethane followed by vacuum treatment at room temperature for 1 hour.

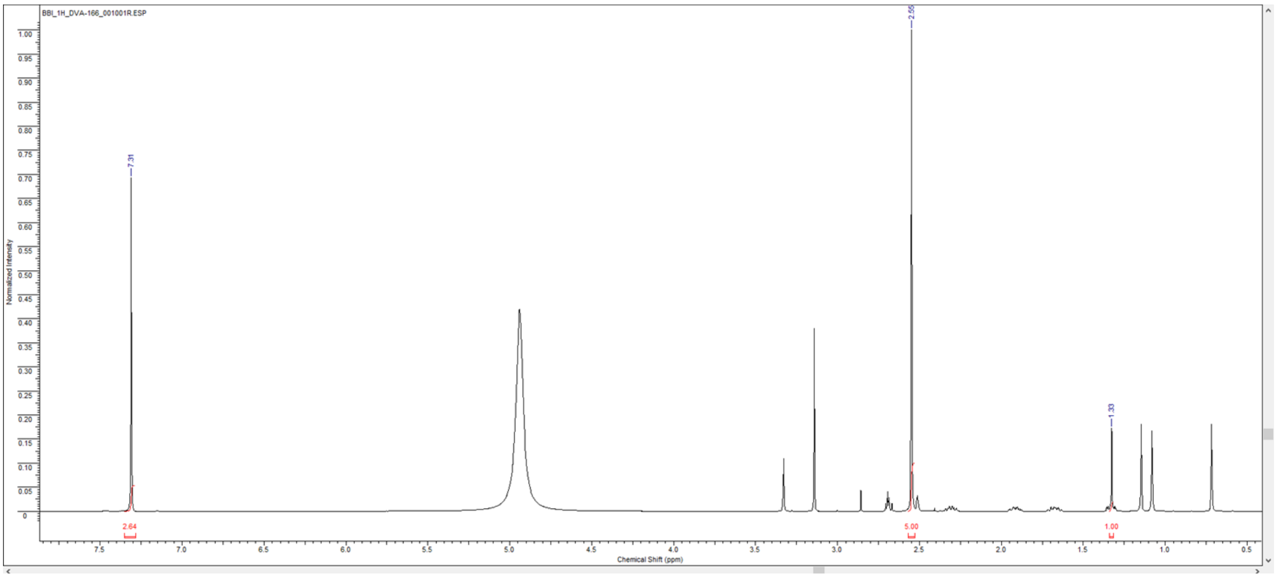

Figure S9. ^{1}H NMR spectrum of benzene and cyclohexane desorbed from the **1-Zn**.

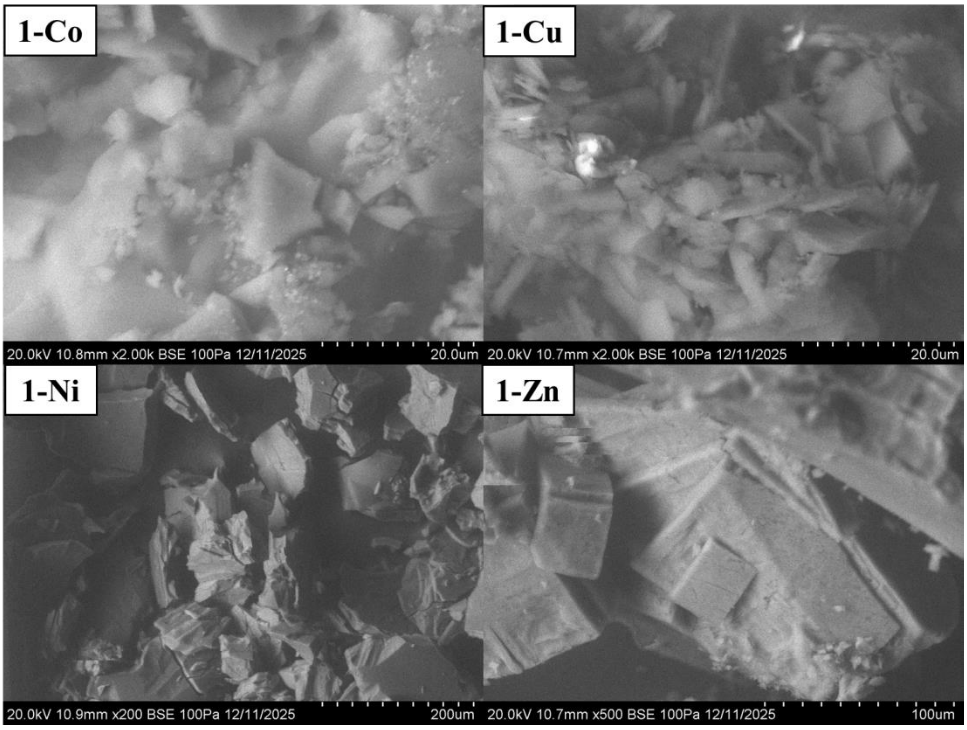

Figure S10. SEM images of the **1-Co, 1-Cu, 1-Ni, 1-Zn** crystals.

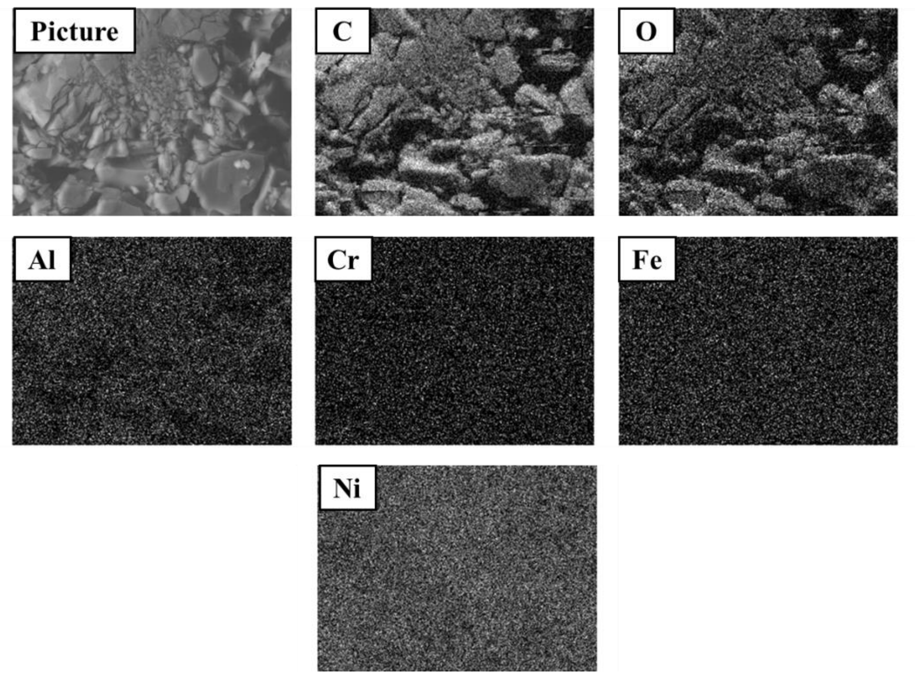

Figure S11. EDS mapping of the **1-Ni** crystals.

Table S2. Elemental composition of **1-Ni, 1-Zn, 1-Co, 1-Cu** determined by energy-dispersive X-ray spectroscopy.

**1-Ni**			**1-Zn**			**1-Co**			**1-Cu**		
Element	wt%	at%	Element	wt%	at%	Element	wt%	at%	Element	wt%	at%
C	58.07	74.36	C	57.90	73.90	C	59.24	74.09	C	57.56	73.84
O	20.83	20.03	O	22.35	21.41	O	22.66	21.27	O	21.84	21.03
Al	0.22	0.12	Al	0.15	0.09	Al	0.21	0.12	Al	0.36	0.20
P	0.02	0.01	Fe	0.21	0.06	Cr	0.21	0.06	Fe	0.53	0.15
Cr	0.15	0.04	Zn	19.39	4.55	Co	15.95	4.07	Cu	19.71	4.78
Fe	0.77	0.21				Zn	1.72	0.39			
Ni	19.95	5.23									

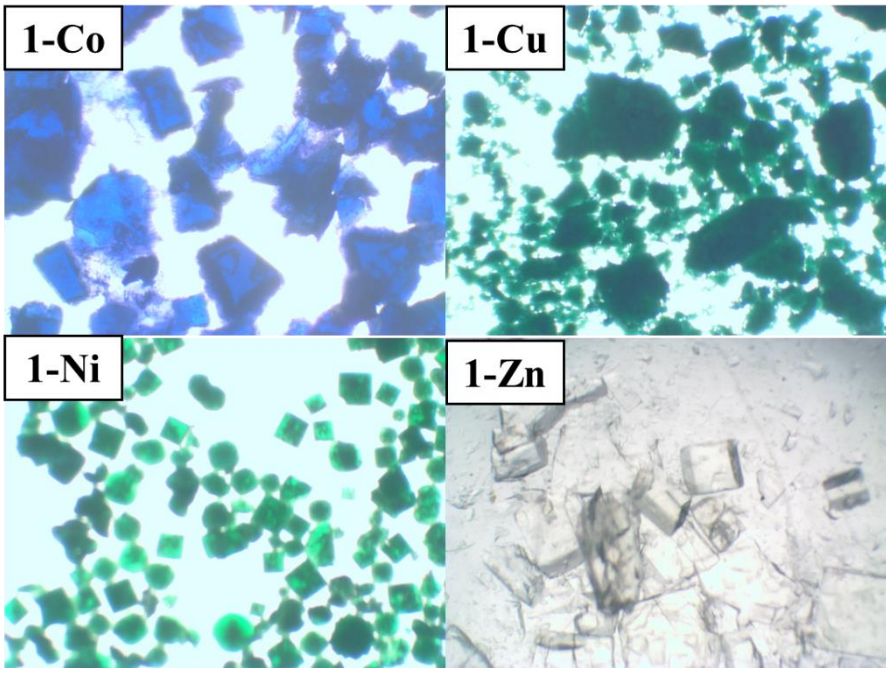

Figure S12. Optical images of the **1-Zn, 1-Ni, 1-Co, 1-Cu** crystals.

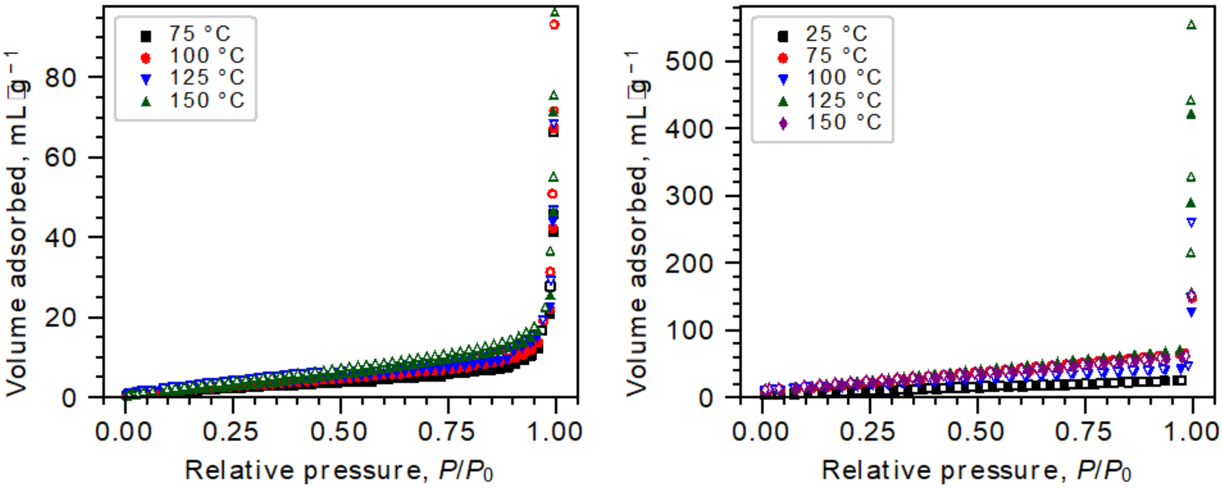

Figure S13. Nitrogen adsorption-desorption isotherms at 77 K for sample **1-Ni** activated from CH_2Cl_2 (left) and THF (right). The corresponding activation temperature is provided in the legend.

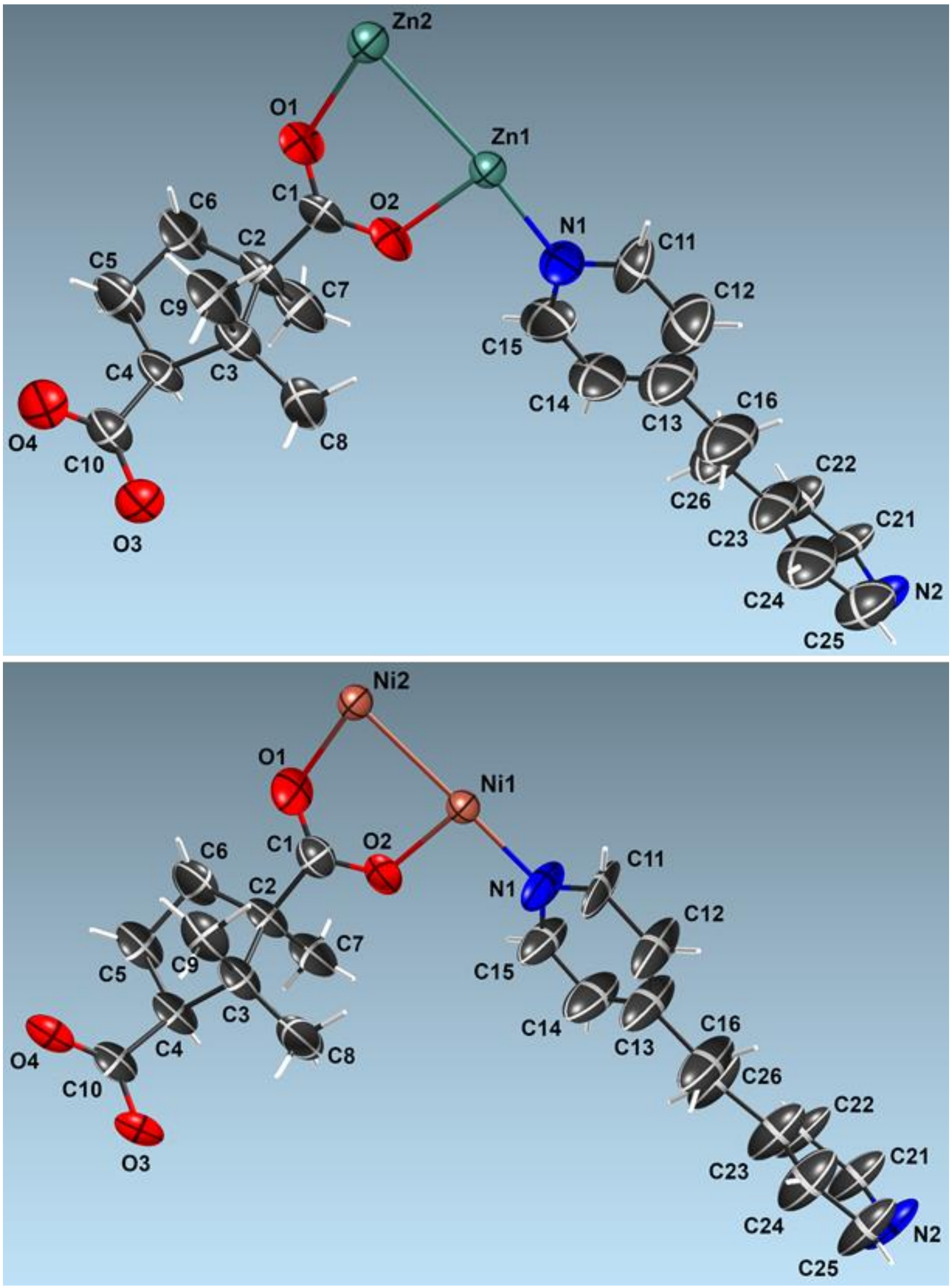

Figure S14. The asymmetric units for compounds **1-Zn** (top) and **1-Ni** (bottom).

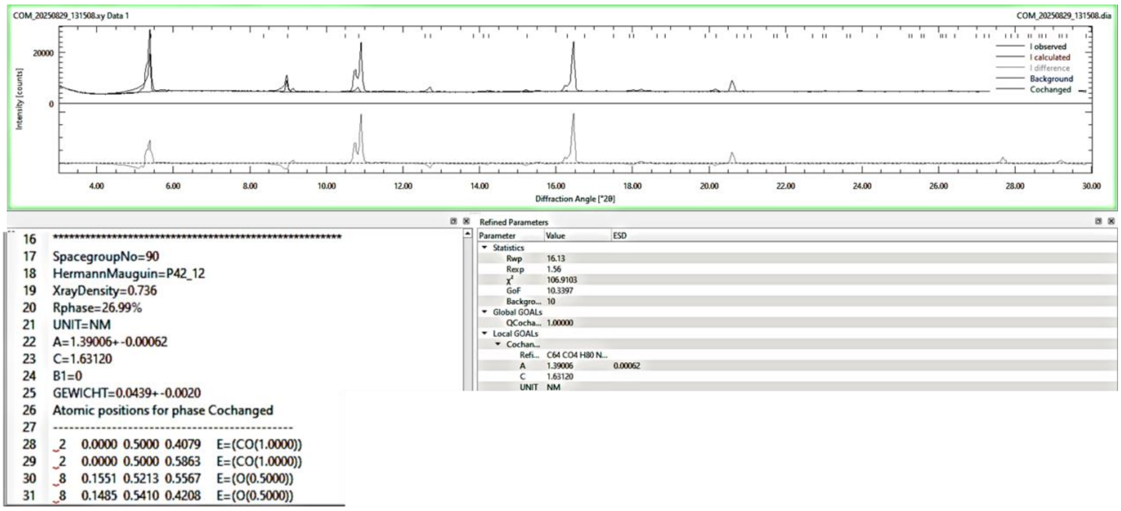

Figure S15. Rietveld refinement of the PXRD data for compound **1-Co** was performed using the Profex software.

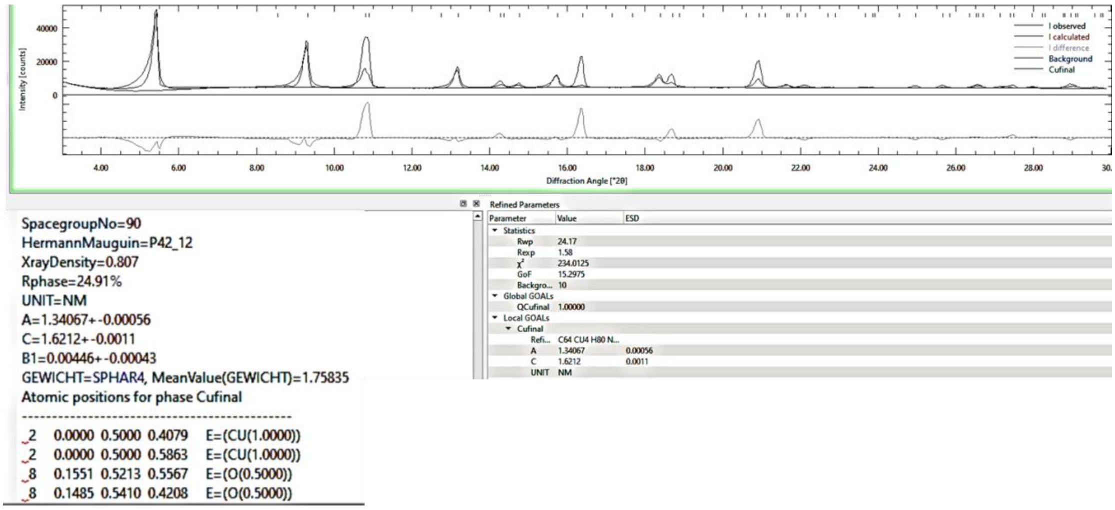

Figure S16. Rietveld refinement of the PXRD data for compound **1-Cu** was performed using the Profex software.

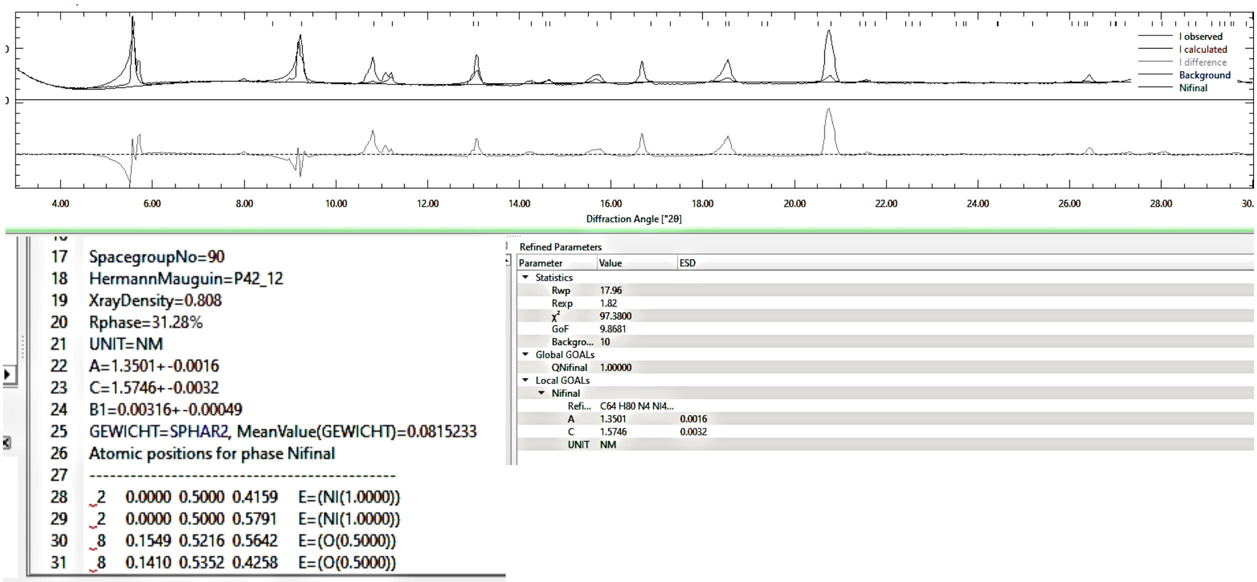

Figure S17. Rietveld refinement of the PXRD data for compound **1-Ni** was performed using the Profex software.

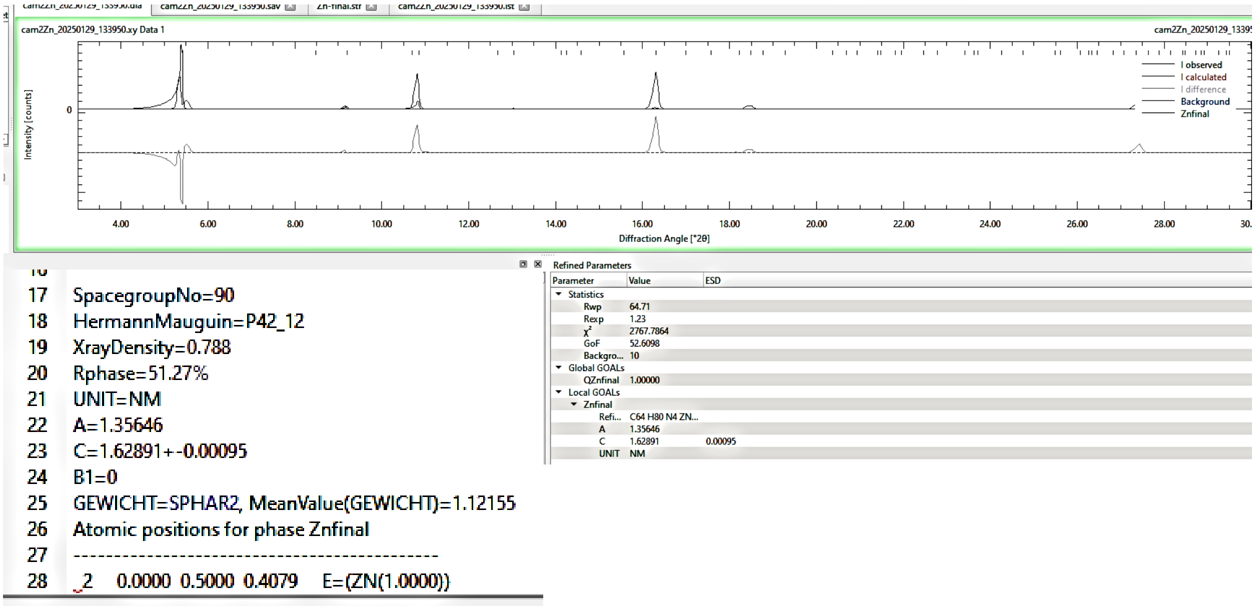

Figure S18. Rietveld refinement of the PXRD data for compound **1-Zn** was performed using the Profex software.
